# Whole-genome sequencing for analysis of an outbreak of meticillin-resistant *Staphylococcus aureus*: a descriptive study

**DOI:** 10.1016/S1473-3099(12)70268-2

**Published:** 2013-02

**Authors:** Simon R Harris, Edward JP Cartwright, M Estée Török, Matthew TG Holden, Nicholas M Brown, Amanda L Ogilvy-Stuart, Matthew J Ellington, Michael A Quail, Stephen D Bentley, Julian Parkhill, Sharon J Peacock

**Affiliations:** a Wellcome Trust Sanger Institute, Cambridge, UK; b Department of Medicine, University of Cambridge, Cambridge, UK; c Department of Pathology, University of Cambridge, Cambridge, UK; d Cambridge Microbiology and Public Health Laboratory, Cambridge, UK; e Cambridge University Hospitals National Health Service Foundation Trust, Cambridge, UK

## Abstract

**Background:**

The emergence of meticillin-resistant *Staphylococcus aureus* (MRSA) that can persist in the community and replace existing hospital-adapted lineages of MRSA means that it is necessary to understand transmission dynamics in terms of hospitals and the community as one entity. We assessed the use of whole-genome sequencing to enhance detection of MRSA transmission between these settings.

**Methods:**

We studied a putative MRSA outbreak on a special care baby unit (SCBU) at a National Health Service Foundation Trust in Cambridge, UK. We used whole-genome sequencing to validate and expand findings from an infection-control team who assessed the outbreak through conventional analysis of epidemiological data and antibiogram profiles. We sequenced isolates from all colonised patients in the SCBU, and sequenced MRSA isolates from patients in the hospital or community with the same antibiotic susceptibility profile as the outbreak strain.

**Findings:**

The hospital infection-control team identified 12 infants colonised with MRSA in a 6 month period in 2011, who were suspected of being linked, but a persistent outbreak could not be confirmed with conventional methods. With whole-genome sequencing, we identified 26 related cases of MRSA carriage, and showed transmission occurred within the SCBU, between mothers on a postnatal ward, and in the community. The outbreak MRSA type was a new sequence type (ST) 2371, which is closely related to ST22, but contains genes encoding Panton-Valentine leucocidin. Whole-genome sequencing data were used to propose and confirm that MRSA carriage by a staff member had allowed the outbreak to persist during periods without known infection on the SCBU and after a deep clean.

**Interpretation:**

Whole-genome sequencing holds great promise for rapid, accurate, and comprehensive identification of bacterial transmission pathways in hospital and community settings, with concomitant reductions in infections, morbidity, and costs.

**Funding:**

UK Clinical Research Collaboration Translational Infection Research Initiative, Wellcome Trust, Health Protection Agency, and the National Institute for Health Research Cambridge Biomedical Research Centre.

## Introduction

Successful prevention of health-care-associated meticillin-resistant *Staphylococcus aureus* (MRSA) depends on effective programmes of infection control, including detection of transmission events and outbreaks and effective strategies for their containment and prevention.^1^, ^2^ Until recently, the predominant focus of control efforts took place in health-care facilities, where most MRSA infections were caused by a small number of health-care-associated MRSA lineages that were poorly adapted for persistence in the community.^3^, ^4^ This situation has undergone a fundamental shift, with the emergence of lineages of community-associated MRSA such as USA300 in the USA that can be carried for long periods by healthy people.^5^, ^6^, ^7^ Furthermore, community-associated MRSA is now displacing previously dominant health-care-associated MRSA lineages,^8^ a process that is driven at least in part by repeated admission and discharge of some groups of patients. One implication of the blurring between health-care-associated and community-associated MRSA is that for the purpose of understanding the transmission dynamics of MRSA, hospitals and the community should no longer be regarded as separate entities. This distinction is problematic, because effective linkage between the two settings is challenging for present strategies used for hospital-based infection control.

One approach to tracking of MRSA transmission pathways between health-care facilities and the community could be to obtain and genotype MRSA isolates, and draw inferences on transmission on the basis of their genetic relatedness. Present typing methods are not fit for purpose because they are not sufficiently discriminatory, but this obstacle is set to change with the introduction of whole-genome sequencing.^9^, ^10^ This technique provides the best discrimination between closely related bacterial isolates, and the rapidly decreasing cost and turnaround time of the technology means that it could become viable in diagnostic laboratories in the near future. Whole-genome sequencing has sufficient discriminatory power to reconstruct intercontinental and local transmission of MRSA lineages.^9^ Furthermore, bench-top, rapid turnaround whole-genome sequencing was able to distinguish between MRSA isolates that were associated with a putative outbreak in a neonatal intensive care unit from those that were not associated with the outbreak.^10^ We proposed that whole-genome sequencing could also be applied to show MRSA transmission between hospitals and the community, and thus emphasise where control strategies could be applied to prevent this occurrence.

We describe the investigation of a new, seemingly restricted MRSA outbreak in our special care baby unit (SCBU), in which a targeted MRSA sequencing strategy was used to detect a previously unsuspected extension of the outbreak into the community and subsequent movement of affected individuals throughout the health-care system. We compared detection of MRSA cases using conventional infection-control methods with targeted whole-genome sequencing in addition to conventional information sources.

## Methods

### Study design and patients

Cambridge University Hospitals NHS Foundation Trust (CUH; Cambridge, UK) is a secondary and tertiary referral hospital with 1000 beds and 67 000 total inpatient admissions, 458 000 visits to outpatients, 115 000 day cases, and 93 000 emergency department attendances every year. The Rosie Hospital is the mother and baby hospital of the Trust, manages about 6000 deliveries every year, and contains a 24 cot SCBU that provides care for infants with acute and chronic neonatal medical and surgical conditions. All infants admitted to this unit are screened for MRSA carriage on admission and once per week thereafter. The onsite clinical microbiology and public health laboratory provides diagnostic microbiology services to the CUH, two additional National Health Service Trusts, and three primary care trusts in the area. About 800 000 clinical specimens are processed every year, 30% of which originate from primary care. In 2011, the laboratory identified nearly 3000 samples as MRSA positive from about 1500 individuals, with more than a third of these samples coming from primary care or outpatient settings. Infection-control management structures, standards, policies, and procedures supporting the prevention and control of infection at CUH are described in our annual report.^11^ Ethical approval was not required for the study because it was done as part of surveillance and management of health-care-associated infection. Research and development approval for whole-genome sequencing was granted by the research and development department at CUH.

### Procedures

We cultured MRSA from screening swabs and clinical specimens by plating on to MRSA selective medium (Brilliance MRSA chromogenic medium, Oxoid, Basingstoke, UK). We identified bacteria with a commercial latex agglutination kit (Pastorex Staph Plus, Bio Rad Laboratories, Hemel Hempstead, UK). We did antimicrobial susceptibility testing with disk diffusion^12^ for the following drugs: cefoxitin, ciprofloxacin, erythromycin, fusidic acid, gentamicin, mupirocin, neomycin, rifampicin, tetracycline, and vancomycin. All isolates were assigned unique strain identification numbers. We prepared sequencing libraries from 500 ng of DNA extracted from each MRSA isolate as previously described,^13^ with amplification using Kapa Hifi polymerase (Kapa Biosystems, Woburn, MA, USA). Whole-genome sequencing was done with an Illumina MiSeq (Illumina, San Diego, CA, USA) to generate 150 bp paired end reads, and interpreted by an investigator (SRH) who was masked to all clinical, epidemiological, and microbiological information. The genome data has been deposited in the European Nucleotide Archive (appendix). We aligned sequence reads to the chromosome (accession number HE681097) and plasmid (CP002148) of a reference isolate (HO 5096 0412) to identify single-nucleotide polymorphisms (SNPs) and insertions or deletions. This reference isolate was defined by multilocus sequence typing as sequence type (ST) 22.

### Role of the funding source

The sponsors of the study had no role in study design, data collection, data analysis, data interpretation, or writing of the report. The corresponding author had full access to all the data in the study and had final responsibility for the decision to submit for publication.

## Results

In 2011, we noted three contemporaneous cases of MRSA carriage on the SCBU at the CUH (patients 11–13; figure A). We regarded these cases as a possible outbreak because of the temporal clustering of the cases and identical (patient 11 and patient 12) or near identical (patient 13) antibiotic susceptibility profiles (appendix). The infection-control team did an investigation in which they completed a systematic review of MRSA isolates from the SCBU during the preceding 6 months. This review identified another 13 infants with one or more positive MRSA screens (patients 1–10 in figure A, and a further three cases not shown). Comparison of the antibiograms (appendix) showed that isolates from eight of the 13 infants (patient 2, patients 4–10, figure A) had an identical pattern to isolates from patient 11 and patient 12, whereas isolates from the five other cases differed by at least two antibiotics and were therefore excluded by the infection control investigation from the suspected outbreak on the basis of usual practice in our hospital (patient 1, patient 3 and the three cases not shown). Thus, 11 infants (patient 2 and patients 4–13) were regarded by the infection-control team to be putatively linked (figure A). Mapping of these 11 cases to a timeline showed that they occurred in three discrete periods, interspersed with gaps of 17 days and 33 days, during which no carriers were detected (figure A). These two gaps meant that the infection-control team was unable to conclude whether a putative outbreak had extended over the 6 months. The recommended actions were to do a deep clean, reinforce infection-control policy and practice, and continue surveillance by weekly MRSA screens. Another infant (patient 14) was detected with carriage of MRSA 4 days after the deep clean was completed, which seemed linked to previous cases on the basis that the antibiogram was identical to patient 11 and patient 12. Overall, the infection-control team assessed 17 cases of MRSA: three cases that triggered the investigation, 13 cases that occurred during the previous 6 months, and one case identified after the deep clean of the SCBU. 12 of these 17 cases were regarded as linked by infection control and five were discounted.

**Figure:**
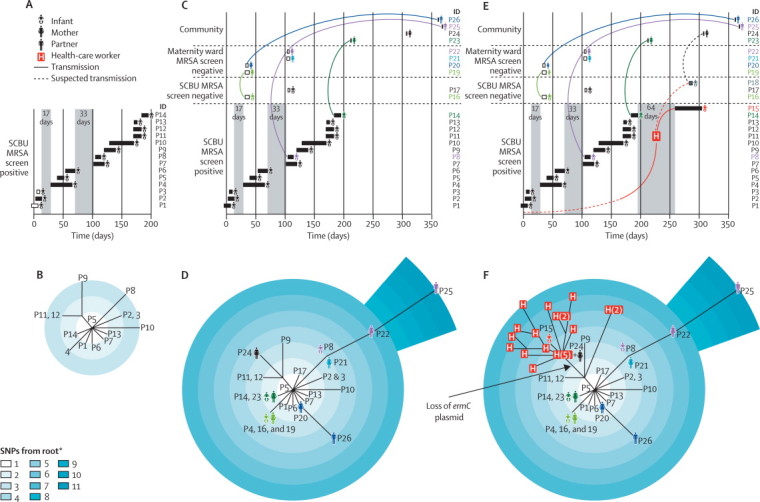
Epidemiology and phylogeny of an outbreak of MRSA sequence type 2371 (A) Epidemiological map of 14 infants on the SCBU (patients 1–14). (B) Phylogenetic tree based on whole-genome sequencing of MRSA isolates from patients 1–14. (C) Epidemiological map of patients 1–14 and ten other patients (patients 16, 17, and 19–26) with linked MRSA infection detected in the community; coloured lines link members of the same family. (D) Phylogenetic tree based on whole-genome sequencing of MRSA isolates from patients 1–14 and patients 16, 17, and 19–26. (E) Epidemiological map of all cases of MRSA identified by whole-genome sequencing, and one patient (patient 18) suspected of being linked to the outbreak but for whom no MRSA colonisation was detected. (F) Phylogenetic tree of all cases of MRSA in the outbreak; 20 individual MRSA colonies from a staff member are shown in red boxes, with multiple colonies from the staff member shown in parentheses. Boxes shown for infants on SCBU in panel A represent duration of hospital stay (black boxes show infants included by the infection-control investigation and white boxes show infants excluded by the infection-control team). Grey vertical blocks in A, C, and E show times on the SCBU when there were no known carriers of MRSA. MRSA=meticillin-resistant *Staphylococcus aureus*. SCBU=special care baby unit. SNP=single-nucleotide polymorphism. P=patient.*Out-group was the sequence type 22 reference genome.

We showed previously that whole-genome sequencing could distinguish between MRSA isolates that are involved in an outbreak and those isolates that are not involved.^10^ Thus, we aimed to retrospectively assess the accuracy of the infection-control investigation of the SCBU outbreak with bacterial sequencing. We anonymised and sequenced the first MRSA isolate from all 17 infants on SCBU who were originally assessed by the infection-control investigation. We used sequencing data to assign each isolate to a sequence type, and noted that 14 isolates were a new sequence type, designated as ST2371 following submission to the multilocus sequence typing website, whereas three other isolates were ST1, ST8, or ST22 (also known as epidemic MRSA-15, which accounts for about 80% of health-care-associated MRSA infection in the UK). ST2371 is a single locus variant of ST22, and differed by one point mutation based on multilocus sequence typing in *arcC*. ST2371 isolates also differed from the ST22 reference in that they possessed the genes coding for Panton-Valentine leucocidin (PVL), which was not identified before sequencing. A phylogenetic tree based on core SNPs identified a unique, highly related cluster made up of all ST2371 isolates (figure B). Only 20 SNPs differentiated these 14 isolates from each other, compared with a mean of 550 SNPs between ST2371 isolates and the reference ST22 genome. In view of the epidemiological data, we regarded this finding as consistent with the involvement of 14 isolates in the SCBU outbreak.

The infection-control team had correctly excluded three isolates (ST1, ST8, and ST22) from the outbreak, but had also incorrectly excluded two isolates (patient 1 and patient 3; figure A) that belonged to the ST2371 clone. This decision had been made because these two isolates differed from the outbreak antibiogram by two antibiotics (appendix). In retrospect, this difference in susceptibility pattern proved to be incorrect because repeat antimicrobial susceptibility testing confirmed these two clones had the same profile as the outbreak. Additionally, whole-genome sequencing suggested that the outbreak spanned the MRSA-free periods on the SCBU.

The infection-control team were not aware of any MRSA transmission events outside of the SCBU, but we hypothesised that MRSA had extended beyond the boundary of the SCBU and used targeted MRSA sequencing to identify MRSA transmission and infection in their contacts. From the hospital microbiology laboratory information system, we identified all MRSA isolates cultured and stored since the start of the putative outbreak. We selected 15 MRSA isolates with an antibiogram that was no more than one antibiotic different from the original outbreak antibiogram (ie, patient 11 and patient 12). We also started prospective surveillance of the microbiology database after identification of patient 14, which yielded four other MRSA isolates with the outbreak antibiogram from non-SCBU samples in the subsequent 3 months. Ten of these 19 isolates were ST2371, five were ST22, and four were ST772. The ST2371 isolates were all closely related to the 14 infant-associated ST2371 isolates from the SCBU (patients 1–14), with only 34 SNPs between all 24 isolates (figure D). Thus, we suspected that the ten new cases might be linked to the SCBU through a previously undetected transmission network.

We identified the source of sample submission for the ten ST2371 isolates as family doctors (six patients), the emergency department at CUH (two patients), and the breast surgery outpatient clinic at the CUH (two patients). We obtained clinical information from the hospital information system to determine whether epidemiological links could be made between these ten cases and the SCBU outbreak. At least one clear link could be made to the SCBU outbreak for nine people (excluding patient 24), with the resulting emergence of a complex network of transmission pathways from infants to their mothers, from mothers-to-mothers in the postnatal ward, and to partners of affected mothers (figure C). Two infants (patient 16 and patient 17) presented to a primary care clinic or the emergency department with an abscess, and had been inpatients on SCBU during the outbreak period when they had been MRSA-screen negative. Two affected mothers (patient 20 and patient 21) did not have infants on SCBU, but did have contact with mothers of MRSA-positive infants on SCBU. One mother with a breast abscess (patient 24) did not seem to have a direct link to a known MRSA carrier, but did have an infant (patient 18) who was an inpatient on SCBU for 5 days (figure E). Her infant had an initial single negative MRSA screen during admission, but we believe that covert MRSA colonisation occurred and was followed by transmission to the mother.

We further examined medical records to assess whether clinical infection was documented in the 24 cases from which MRSA ST2371 was isolated. 14 of these patients had documented clinical infection (table), four while in the SCBU. Five mothers had breast abscesses, one of which was associated with cellulitis and tissue necrosis. The predominance of skin and soft-tissue infections we noted is consistent with the finding that ST2371 encodes PVL. All individuals were treated with MRSA decolonisation therapy and all ten patients with an abscess received systemic antibiotics.

**Table:** Clinical infections and transmission of MRSA sequence type 2371

	**Description**	**Type of infection**	**Presentation**	**Treatment**
Patient 1	Infant (part of outbreak)	Superficial pustules	Inpatient	MRSA decolonisation therapy
Patient 2	Infant (part of outbreak)	Superficial pustules	Inpatient	MRSA decolonisation therapy
Patient 4	Infant (part of outbreak)	Superficial pustules	Inpatient	MRSA decolonisation therapy
Patient 11	Infant (part of outbreak)	Superficial pustules	Inpatient	MRSA decolonisation therapy
Patient 16	Infant on SCBU for 5 days during outbreak, negative MRSA screen	Abscess (chest wall)	13 days after discharge from SCBU	One ED visit, one hospital outpatient clinic visit, four primary care visits, MRSA decolonisation therapy
Patient 17	Infant, on SCBU for 3 days during outbreak, negative MRSA screen	Abscess (cheek)	29 days after discharge from SCBU	One primary care visit, MRSA decolonisation therapy
Patient 19	Mother of patient 16 (a known MRSA carrier on SCBU)	Abscess (breast)	21 days after infant discharged from SCBU	Two breast surgery clinic visits, one ED visit, two primary care visits, MRSA decolonisation therapy
Patient 20	Mother of infant not on SCBU; contact in postnatal ward with mothers of MRSA positive infants in SCBU	Abscess (breast)	17 days after discharged home following delivery	Four breast surgery clinic visits, one ED visit, one primary care visit, 3 days of inpatient treatment, MRSA decolonisation therapy
Patient 21	Mother of infant not on SCBU; contact in postnatal ward with mothers of MRSA-positive infants in SCBU	Abscess (breast)	26 days after discharged home following delivery	Four breast surgery clinic visits, one ED visit, one primary care visit, MRSA decolonisation therapy
Patient 22	Mother of infant patient 8 (known MRSA carrier on SCBU)	Abscess (thigh)	175 days after MRSA carriage detected in own infant	One primary care visit, MRSA decolonisation therapy
Patient 23	Mother of infant patient 14 (known MRSA carrier on SCBU)	Abscess (breast)	18 days after MRSA carriage detected in own infant	One ED visit, one breast surgery clinic visit, MRSA decolonisation therapy
Patient 24	Mother of patient 18 (MRSA-screen negative infant on SCBU)	Abscess (breast)	11 days after own infant was discharged from SCBU	Four breast surgery clinic visits, MRSA decolonisation therapy
Patient 25	Partner of patient 22 and father of infant patient 8	Abscess (ear)	257 days after MRSA carriage detected in infant, and 82 days since partner presented with abscess on thigh	One primary care visit, one ED visit, three hospital outpatient clinic visits, 1 day of inpatient treatment, MRSA decolonisation therapy
Patient 26	Partner of patient 20	Abscess (thigh)	325 days after mother and infant discharged home after delivery	One primary care visit, MRSA decolonisation therapy

MRSA=meticillin-resistant *Staphylococcus aureus*. SCBU=special care baby unit. ED=emergency department.

Prospective longitudinal surveillance for MRSA carriage on the SCBU led us to identify a new MRSA carrier (patient 15) who was admitted 64 days after the previous MRSA-positive patient left the unit (figure E). We sequenced this isolate immediately on the MiSeq platform, with a culture to sequence turnaround of 48 h,^10^ and noted that the isolate was ST2371 with only four SNPs different from the genetically closest SCBU outbreak isolate (figure F). We noted that there was only one SNP difference between the MRSA isolates from patient 15 and patient 24, suggesting a possible transmission pathway (figure E). The re-emergence of ST2371 on the SCBU after the deep clean led us to suspect that one or more members of staff might be carrying and transmitting the outbreak MRSA strain. This notion was supported by the structure of the phylogenetic tree for the SCBU outbreak isolates, which did not show a strong temporal signature of sequential patient transmission, but suggested that repeated introduction from an external source was possible. The combined whole-genome sequencing and epidemiological data were presented at a meeting of the infection-control team, senior clinicians, nurses, and managers on the SCBU, which resulted in the decision to screen staff members. After obtaining written informed consent, the infection-control team screened 154 SCBU staff members for MRSA colonisation. One staff member was positive for MRSA, which was confirmed as ST2371 with whole-genome sequencing. The staff member was relieved from clinical duties and reviewed by the occupational health department. The staff member had no skin lesions and underwent successful MRSA decolonisation.

We postulated that if the staff member had been linked to the outbreak, we might discover a genetic record by sequencing numerous colonies from the staff member and matching these with isolates from the infants. 20 MRSA colonies were isolated from the original swab taken from the staff member and sequenced. When included in a phylogenetic tree with the isolates from the infants, we noted a cloud of diversity for the 20 staff colonies, including strains that were a close genetic match to isolates from infants before and after the most recent MRSA-free gap of 64 days. In particular, 18 colonies from the staff member clustered with patient 15, who was the last infant to be colonised with MRSA in the SCBU. The other two colonies clustered closer towards the root of the tree (figure F). From the number of SNPs between the staff MRSA colonies and a previously calculated mutation rate for ST22 of about one SNP per 15 weeks (MTG Holden, Wellcome Trust Sanger Institute, Cambridge, UK, personal communication), we calculated a mean estimate for the time that the staff member was colonised of 23 days before the first MRSA positive infant (patient 1) was detected (figure E), and a range of 251 days before to 164 days after the first case. Further evidence for transmission by the staff member either side of the MRSA-free gap came from the observation that a genetic change had occurred in 18 of 20 colonies involving loss of a plasmid that carried *ermC* (encoding erythromycin resistance). This genetic and corresponding phenotypic change was also reported for two other isolates, from the last infant carrier, patient 15, and from patient 24, whose child (patient 18) was on SCBU at the same time as patient 15 (figure F).

## Discussion

In our study, more precise identification of patients involved in an outbreak of MRSA was possible with whole-genome sequencing than with standard infection-control techniques (panel). Moreover, use of bacterial whole-genome sequencing in real time was able to identify the potential source of an ongoing MRSA outbreak and directly inform infection-control interventions. A conservative estimate of the UK National Health Service health-care costs attributable to the outbreak in our hospital was in excess of £10 000. By comparison, the cost of rapid whole-genome sequencing of one MRSA isolate is £95 at present, including sample preparation, library quality control, and sequencing.

Panel **Research in context**

**Systematic review**

We searched PubMed without language restriction up to Nov 6, 2012, with the search terms “whole-genome sequencing” AND “MRSA” AND “outbreak”. We identified two studies^10^, ^14^ in which whole-genome sequencing was used to retrospectively sequence clinical isolates of meticillin-resistant *Staphylococcus aureus* (MRSA). These studies confirmed that whole-genome sequencing clustered isolates associated with outbreaks,^10^, ^14^ and that sequence data were concordant with antimicrobial susceptibility testing and could be used to create an inventory of toxin genes.^10^ We identified no reports on the assessment of whole-genome sequencing to detect MRSA transmission networks between hospitals and the community. This absence of data is notable because MRSA control needs to bridge the gap between the two settings, and effective linkage between the hospital and community is challenging for current strategies used by infection control.

**Interpretation**

Addition of whole-genome sequencing to an infection-control investigation of an MRSA outbreak in a special care baby unit allowed enhanced case detection and identification of an MRSA transmission network in our study, which extended into the community. Routine real-time use of whole-genome sequencing could have detected the outbreak 6 months earlier than identification based on clustering of cases, and might have prevented morbidity and costs resulting from MRSA transmission and subsequent infection. Development of techniques that provide automated analysis of sequence data and cost-benefit analyses of the use of this technology in the control of MRSA are needed.

The outbreak we investigated was chosen because standard approaches had already obtained information against which we could compare whole-genome sequencing, but it was otherwise unremarkable. Whole-genome sequencing of isolates with the same antibiogram collected within a year after the putative start of the outbreak identified ten previously unlinked cases of MRSA skin and soft-tissue infections occurring outside of the inpatient setting. These cases corresponded with transmission events between infants and their parents, and onward transmission between mothers in a postnatal ward, and were associated with substantial human morbidity and health-care costs. Three factors might have hampered the ability of the routine infection-control processes to detect transmissions: first, clusters of MRSA carriers in the SCBU were separated by periods when no MRSA carriers were detected; second, some cases presented in primary care or outpatient settings; and third, a substantial delay existed between the initial outbreak investigation and presentation of some of the community cases. Our findings probably represent the minimum number involved in the outbreak. Access to the microbiology database increased our capture of possible cases, but MRSA transmission to other individuals who did not present with infection could have eluded detection. This possibility is supported by reports that several infants who developed MRSA infection after discharge from hospital were MRSA screen negative on their initial screen in the SCBU, but presumably acquired MRSA before going home. This notion is plausible because routine MRSA screens were done once per week.

We showed that the SCBU cases were linked despite the absence of an obvious ongoing transmission chain, and rapidly noted a new case of MRSA 64 days after the last MRSA-positive patient had left the unit. This sequence of events suggested reintroduction from a carrier, who was identified as one of the staff members. Such rapid identification provides a practical application of whole-genome sequencing in a clinically relevant timeframe, which is associated with an intervention that reduced the risk of further transmission, infection, and associated morbidity.

Sequencing of several colonies from the staff member showed that they were carrying many variants of the outbreak strain, as would be expected after prolonged carriage. We did not generate the sequence of multiple isolates from other patients, but in most cases the amount of time that elapsed between the putative time of acquisition and the point of sampling would not have been long enough to generate substantial variation in view of the known rate of mutation in the *S aureus* genome. That colonies carried by the staff member were segregated into those that matched most closely to isolates from infants before the 64 day MRSA-free gap and those after the gap suggests strongly that this staff member had reintroduced the strain to the SCBU after the deep clean. However, we cannot be certain that the same staff member was responsible for transmission across the earlier MRSA-free periods.

Whole-genome sequencing also showed the unexpected identification of a novel clone of MRSA that is closely related to the predominant health-care-associated MRSA clone in the UK, epidemic MRSA-15. Unlike the pattern of MRSA infection in the USA associated with the emergence of USA300, in which community-associated infection predominates and is associated with skin and soft-tissue infection, epidemic MRSA-15 is mainly associated with health-care-associated transmission and is not associated with extensive community transmission or skin and soft-tissue infections. The biological behaviour of the novel ST seems to differ from epidemic MRSA-15 and is comparable with USA300,^5^, ^15^, ^16^, ^17^ with transmission between family members, prolonged carriage, and increased rates of skin and soft-tissue infections.

Health-care-associated MRSA is not routinely genotyped in the UK because most cases belong to a single clone (ST22), and so individual isolate types cannot be distinguished. The use of multilocus sequence typing during the investigation described here would have shown that the outbreak was due to a new clone, but a shared multilocus sequence type alone would not have been definitive proof that the sequenced isolates were all from one outbreak. Moreover, further testing would have been needed to show the presence of the gene coding for PVL. The time taken to detect this gene and perform multilocus sequence typing, including repeat sequencing to verify a new point mutation in *arcC*, would outweigh the time taken to do one sequencing run. In this study, isolates from patient 15 and patients 23–26 inclusive were prospectively sequenced with a sequencing run that took 1 day to complete and a bioinformatics pipeline that required less than 1 h of analysis time.

Whole-genome sequencing of MRSA could make an important contribution to infection-control investigation and practice. Rapid sequencing would have confirmed the outbreak on SCBU close to its start point, and routine whole-genome sequencing of MRSA from all new cases identified in the microbiology laboratory would have rapidly drawn links between the SCBU and the community. Implementation of routine whole-genome sequencing for MRSA would need the development of a central database for comparison of sequence data with previous local, national, and global isolates, and development of a system for automated interpretation and linking of genome sequence data. Nevertheless, such an approach holds great promise for rapid, accurate, and comprehensive identification of bacterial transmission pathways in hospital and community settings, with concomitant reductions in infections, morbidity, and costs.
