# Supplementary material for: Whole-genome sequencing for analysis of an outbreak of meticillin-resistant Staphylococcus aureus: a descriptive study
Source: Lancet Infect Dis. 2013 Feb;13(2):130–6. doi: 10.1016/S1473-3099(12)70268-2 (PMC3556525; doi:10.1016/S1473-3099(12)70268-2)
Supplement: Supplementary appendix [file mmc1.pdf]

THE LANCET Infectious Diseases

Supplementary webappendix

This webappendix formed part of the original submission and has been peer reviewed. We post it as supplied by the authors.

Supplement to: Harris SR, Cartwright EJP, Török ME, et al. Whole-genome sequencing for analysis of an outbreak of meticillin-resistant *Staphylococcus aureus*: a descriptive study. *Lancet Infect Dis* 2012; published online Nov 14. http://dx.doi.org/10.1016/S1473-3099(12)70268-2.

Supplement for 'Whole-genome sequencing for analysis of an outbreak of meticillin-resistant Staphylococcus aureus: a descriptive study'

Simon R. Harris PhD, Edward J.P. Cartwright MBBS, M. Estée Török FRCP, Matthew T.G. Holden PhD, Nicholas M. Brown FRCPath, Amanda L. Ogilvy-Stuart FRCP, Matthew J. Ellington DPhil, Michael A. Quail PhD, Stephen D. Bentley PhD, Prof. Julian Parkhill PhD, Prof. Sharon J. Peacock FRCP

METHODS

Mapping of sequenced data and variation detection were carried out using an automated in-house pipeline.

Mapping

Sequenced reads were mapped against the ST22 reference (HO 5096 0412) using SMALT v 0.5.8 (http://www.sanger.ac.uk/resources/software/smalt/) to produce bam files. Reads were only mapped if they matched the reference with an identity of greater than 90%.

Indel identification and realignment

As mapping programmes align each read in isolation, insertion and deletion (indel) positions in reads relative to the reference sequence are not always consistently aligned across all reads. This can lead to misidentification of variation around indels. To reduce this potential error the pipeline assesses the evidence for indels across the genome and, once strongly supported indels are identified, realigns the reads in their vicinity. First, candidate indels relative to the reference were identified with pindel.[1] For each candidate indel, relevant reads (those mapping within 1 read length of the indel and those not mapping against the reference sequence in the initial mapping) were remapped against a version of the genome with the proposed indel included, and mapping statistics compared to that without the indel. Where multiple indels were found within a read length, combinations of presence and absence of each indel were compared. Where the inclusion of an indel improved mapping, the indel was accepted. A final mapping was completed against a version of the reference including all accepted indels, to produce a bam file whose co-ordinates were adjusted to fit the original reference sequence.

Variation detection

Variation statistics at each base were calculated using samtools mpileup followed by bcftools view from the samtools package.[2] All bases were then filtered to remove those with uncertainty in the base call. The bcftools variant quality score was required to be greater than 50 and mapping quality greater than 30. If not all reads gave the same base call, the allele frequency, as calculated by bcftools, was required to be either 0 for bases called the same as the reference, or 1 for bases called as a SNP. The majority base call was required to be present in at least 80% of reads mapping at the base, and the minimum mapping depth

allowed was 4 reads, at least two of which had to map to each strand. If any of these filters were not met, the base was called as uncertain.

Phylogenetic analysis

Phylogenetic reconstruction was carried out using the maximum-likelihood programme RAxML v 7.0.4[3] with a GTR model of evolution. SNPs were reconstructed onto the tree using the deltran parsimony criterion using a script written in biopython.

References

1. Ye, K., Schulz, M. H., Long, Q., Apweiler, R. & Ning, Z. Pindel: a pattern growth approach to detect break points of large deletions and medium sized insertions from paired-end short reads. *Bioinformatics* **25**, 2865–2871 (2009).
2. Li, H. *et al.* The Sequence Alignment/Map format and SAMtools. *Bioinformatics* **25**, 2078–2079 (2009).
3. Stamatakis, A. RAxML-VI-HPC: maximum likelihood-based phylogenetic analyses with thousands of taxa and mixed models. *Bioinformatics* **22**, 2688–2690 (2006).

Table S1. Antimicrobial susceptibility pattern of MRSA isolates.

ID	Cefoxitin	Ciprofloxacin	Erythromycin	Fusidic Acid	Gentamicin	Mupirocin	Rifampicin	Tetracycline	Vancomycin	Neomycin
H (Healthcare worker)	Red	Red	Blue	White	Red	White	White	White	White	Red
26 (Partner)	Red	Red	Red	White	Red	White	White	White	White	Red
25 (Partner)	Red	Red	Red	White	Red	White	White	White	White	Red
24 (Mother)	Red	Red	White	White	Red	White	White	White	White	Red
23 (Mother)	Red	Red	Red	White	Red	White	White	White	White	Red
22 (Mother)	Red	Red	Red	White	Red	White	White	White	White	Red
21 (Mother)	Red	Red	Red	White	Red	White	White	White	White	Red
20 (Mother)	Red	Red	Red	White	Red	White	White	White	White	Red
19 (Mother)	Red	Red	Red	White	Red	White	White	White	White	Red
17 (Infant)	Red	Red	Red	White	Red	White	White	White	White	Red
16 (Infant)	Red	Red	Red	White	Red	White	White	White	White	Red
15 (Infant)	Red	Red	White	White	Red	White	White	White	White	Red
14 (Infant)	Red	Red	Red	White	Red	White	White	White	White	Red
13 (Infant)	Red	Red	Red	White	Red	White	White	White	White	Green
12 (Infant)	Red	Red	Red	White	Red	White	White	White	White	Red
11 (Infant)	Red	Red	Red	White	Red	White	White	White	White	Red
10 (Infant)	Red	Red	Red	White	Red	White	White	White	White	Red
9 (Infant)	Red	Red	Red	White	Red	White	White	White	White	Red
8 (Infant)	Red	Red	Red	White	Red	White	White	White	White	Red
7 (Infant)	Red	Red	Red	White	Red	White	White	White	White	Red
6 (Infant)	Red	Red	Red	White	Red	White	White	White	White	Red
5 (Infant)	Red	Red	Red	White	Red	White	White	White	White	Red
4 (Infant)	Red	Red	Red	White	Red	White	White	White	White	Red
3 (Infant)	Red	Red	Red	White	Green	White	White	White	White	Green
2 (Infant)	Red	Red	Red	White	Red	White	White	White	White	Red
1 (Infant)	Red	Red	Red	White	Green	White	White	White	White	Green

Key:

Infant

Mother

Partner

Healthcare worker

Red means resistant and white means susceptible to a given antibiotic. Green, initial result proved incorrect on repeat testing and changed designation from susceptible to resistant. A healthcare worker had twenty MRSA colonies taken from the primary plate; the blue box for erythromycin signifies that some colonies were susceptible to this antibiotic (n=18) and some were resistant (n=2).

Table S2. Accession numbers of genome sequence data for each MRSA isolate. Deposited at the European Nucleotide Archive (ENA, http://www.ebi.ac.uk/ena/).

Name in paper	MLST	Accession number	Involved in outbreak?
P1	ST2371	ERR070045	Yes
P2	ST2371	ERR070042	Yes
P3	ST2371	ERR070043	Yes
P4	ST2371	ERR070044	Yes
P5	ST2371	ERR070046	Yes
P6	ST2371	ERR070047	Yes
P7	ST2371	ERR070048	Yes
P8	ST2371	ERR070033	Yes
P9	ST2371	ERR070034	Yes
P10	ST2371	ERR070036	Yes
P11	ST2371	ERR070039	Yes
P12	ST2371	ERR070040	Yes
P13	ST2371	ERR070038	Yes
P14	ST2371	ERR072246	Yes
P15	ST2371	ERR108054	Yes
P16	ST2371	ERR124429	Yes
P17	ST2371	ERR124430	Yes
P19	ST2371	ERR124432	Yes
P20	ST2371	ERR124431	Yes
P21	ST2371	ERR124433	Yes
P22	ST2371	ERR124434	Yes
P23	ST2371	ERR072247	Yes
P24	ST2371	ERR124435	Yes
P25	ST2371	ERR128708	Yes
P26	ST2371	ERR128707	Yes
Healthcare worker, MRSA colonies 1-20 picked from primary culture plate inoculated with MRSA screening swab	ST2371	ERR128709	Yes
	ST2371	ERR128710	
	ST2371	ERR128711	
	ST2371	ERR128712	
	ST2371	ERR131808	
	ST2371	ERR131809	
	ST2371	ERR131810	
	ST2371	ERR131811	
	ST2371	ERR131812	
	ST2371	ERR131813	
	ST2371	ERR131814	
	ST2371	ERR131815	
	ST2371	ERR128713	
	ST2371	ERR128714	
	ST2371	ERR128715	
	ST2371	ERR128716	
	ST2371	ERR128717	
	ST2371	ERR128718	

	ST2371	ERR128719	
	ST2371	ERR128720	
Identified by Infection Control investigation, non-outbreak	ST1	ERR070041	No
	ST8	ERR070035	No
	ST22	ERR070037	No
MRSA identified by searching microbiology database and based on criterion that the antibiogram was no more than one antibiotic different to the MRSA outbreak antibiogram (P11/P12)	ST772	ERR072248	No
	ST772	ERR124436	No
	ST772	ERR131800	No
	ST772	ERR131804	No
	ST22	ERR131801	No
	ST22	ERR131802	No
	ST22	ERR131805	No
	ST22	ERR131806	No
	ST22	ERR131807	No
Reference MRSA isolated used for mapping (strain number HO 5096 0412)	ST22	HE681097	No
